# Supplementary material for: TruePrime is a novel method for whole-genome amplification from single cells based on TthPrimPol
Source: Nat Commun. 2016 Nov 29;7:13296. doi: 10.1038/ncomms13296 (PMC5141293; doi:10.1038/ncomms13296)
Supplement: Supplementary Information — Supplementary Figures 1-8, Supplementary Table 1 and Supplementary References [file ncomms13296-s1.pdf]

Supplementary Figures

Supplementary Figure 1

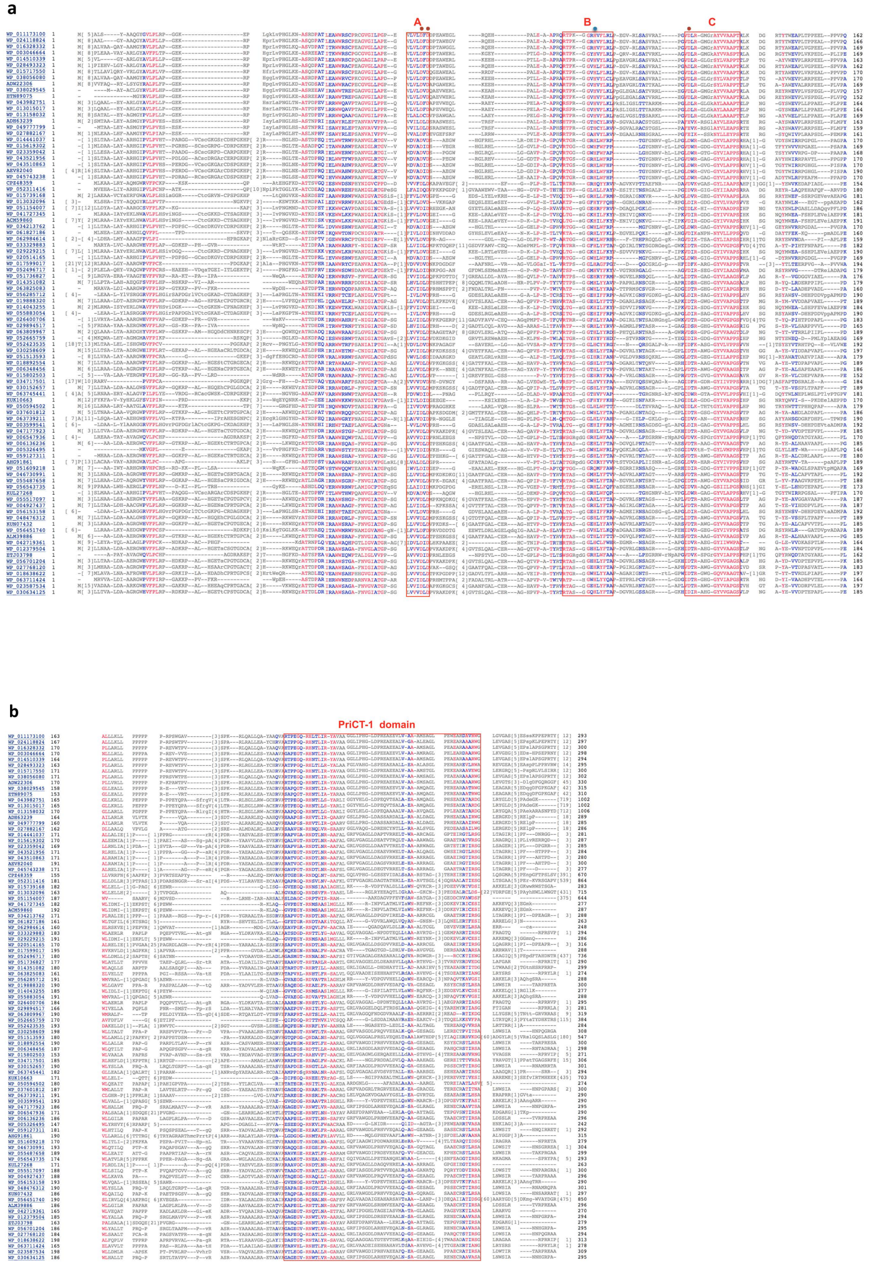

Phylogenetic tree of TthPrimPol and its closest bacterial relatives. The amino acid sequence of TthPrimPol (WP_011173100.1) from Thermus thermophilus HB27 was used to scrutinize the non-redundant (NR) database of protein sequences (National Center for Biotechnology Information, NIH, Bethesda), using the BLASTP program[1]. (**a and b**) By exclusively searching among Bacteria, the first 90 highest scores were selected, and their amino acid sequences were aligned by using the multiple

alignment tool provided at the BLAST server, using the following alignment, CDD and query clustering parameters.: gap penalties (-11, -1); end-gap penalties (-5, -1); blast E-value: (0.003); word size: (4); max. cluster distance: (0.8). The multiple alignment confirmed the presence of extensive similarities, in addition to the three most conserved AEP motifs (A, B and C), that contain the three invariant catalytic carboxylates (indicated with a red dot in motifs A and C) and the invariant histidine (indicated with a blue dot in motif B), and the C-terminal PriCT-1 domain[2].

WP_011173100.1 *Thermus thermophilus* HB27;
WP_024118824.1 *Thermus thermophilus sp.*;
WP_016328332.1 *Thermus oshimai*;
WP_003046664.1 *Thermus aquaticus;*
WP_014510339.1 *Thermus thermophilus* SG0.5JP17-16;
WP_028493323.1 *Thermus antranikianii*;
WP_015717550.1 *Thermus scotoductus*;
WP_038056080.1 *Thermus amyloliquefaciens*;
ADW22306.1 *Thermus scotoductus* SA-01;
WP_038029545.1 *Thermus sp*. NMX2.A1;
ETN89075.1 *Thermus sp.* NMX2.A1;
WP_043982751.1 *Meiothermus ruber*;
WP_013015017.1 *Meiothermus ruber*;
WP_013158032.1 *Meiothermus silvanus*;
ADH63239.1 *Meiothermus silvanus* DSM 9946;
WP_049777799.1 *Meiothermus silvanus*;
WP_027882167.1 *Meiothermus rufus*;
WP_014441037.1 *Actinoplanes missouriensis*;
WP_015619302.1 *Actinoplanes sp.* N902-109;
WP_023359042.1 *Actinoplanes friuliensis*;
WP_043521956.1 *Actinoplanes utahensis*;
WP_043510863.1 *Actinoplanes sp*. SE50/110;
AEV82040.1 *Actinoplanes sp.* SE50/110;
WP_045743238.1 *Actinoplanes rectilineatus*;
CPZ48359.1 *Mycobacterium abscessus*;
WP_052311416.1 *Methylobacterium sp*. GXF4;
WP_015739168.1 *Ammonifex degensii*;
WP_013032096.1 *Nitrosococcus halophilus*;
WP_051154007.1 *Burkholderia sp*. URHA0054;
WP_041727345.1 *Caldicellulosiruptor bescii*;
ACM59860.1 *Caldicellulosiruptor bescii* DSM 6725;
WP_034213762.1 *Actinoplanes subtropicus*;
WP_061827186.1 *Lactobacillus sakei*;
WP_062984614.1 *Nocardia anaemiae*;
WP_033329883.1 *Actinomadura madurae*;
WP_029229215.1 *Caldicellulosiruptor acetigenus*;
WP_020514165.1 *Actinoplanes globisporus*;
WP_017599017.1 *Nocardiopsis lucentensis*;
WP_052496717.1 *Agrobacterium tumefaciens*;
WP_051736827.1 *Pseudonocardia autotrophica*;
WP_014351082.1 *Nocardia cyriacigeorgica*;
WP_063825083.1 *Nocardia pseudovaccinii*;
WP_056285712.1 *Methylobacterium sp*. Leaf108;
WP_019888320.1 *Streptomyces purpureus*;
WP_014043255.1 *Caldicellulosiruptor lactoaceticus*;
WP_055883054.1 *Methylobacterium sp.* Leaf399;
WP_026400706.1 *Actinomadura rifamycini*;
WP_029894517.1 *Nocardia*;
WP_063809967.1 *Kibdelosporangium phytohabitans*;
WP_052665759.1 *Clostridium sp.* FS41;
WP_052423535.1 *Nonomuraea candida*;
WP_030258609.1 *Streptomyces violens*;
WP_051513593.1 *Skermanella stibiiresistens*;
WP_018892554.1 *Streptomyces sp*. CNT302;
WP_006348456.1 *Streptomyces tsukubensis*;
WP_015802503.1 *Actinosynnema mirum*;
WP_034717501.1 *Intrasporangium chromatireducens*;
WP_030152657.1 *Streptomyces sp*. NRRL S-244;
WP_063745441.1 *Dactylosporangium aurantiacum*;
KUK10663.1 *Clostridia bacterium* 41_269;
WP_050594502.1 *Mycobacterium avium*;
WP_037601812.1 *Streptacidiphilus rugosus*;
WP_063739211.1 *Streptomyces hokutonensis*;
WP_003599541.1 *Methylobacterium extorquens*;
WP_047177923.1 *Streptomyces sp.* MNU77;
WP_006547936.1 *Actinomyces urogenitalis*;
WP_006136236.1 *Streptomyces gancidicus;*
WP_005326495.1 *Corynebacterium tuberculostearicum*;
WP_059127311.1 *Streptomyces sp.* NRRL F-5122;
AHG91861.1 *Gemmatirosa kalamazoonesis*;

WP_051609218.1 *Fodinicurvata fenggangensis*;
WP_046730991.1 *Streptomyces sp*. MUSC119T;
WP_055487658.1 *Streptomyces sp*. WMMB 322;
WP_056543735.1 *Rhizobium sp*. Root1220;
KUL27268.1 *Actinoplanes awajinensis* subsp. *mycoplanecinus*;
WP_055517097.1 *Streptomyces aurantiacus;*
WP_004927437.1 *Streptomyces griseoflavus*;
WP_056153158.1 *Methylobacterium sp.* Leaf92;
WP_048476312.1 *Streptomyces roseus*;
KUN07432.1 *Streptomyces yokosukanensis*;
WP_056451740.1 *Sphingomonas sp.* Leaf10;
ALM39886.1 *Streptomyces sp.* FR-008;
WP_042719361.1 *Komagataeibacter europaeus*;
WP_012379504.1 *Streptomyces griseus*;
ETJ03798.1 *Actinomyces urogenitalis* DORA_12;
WP_056701204.1 *Streptomyces*;
WP_027768120.1 *Streptomyces sp*. CNQ865;
WP_018638622.1 *Frankia sp*. BMG5.12;
WP_063711424.1 *Nocardia asiatica*;
WP_023587534.1 *Streptomyces thermolilacinus*;
WP_030634125.1 *Streptomyces albus*.

c

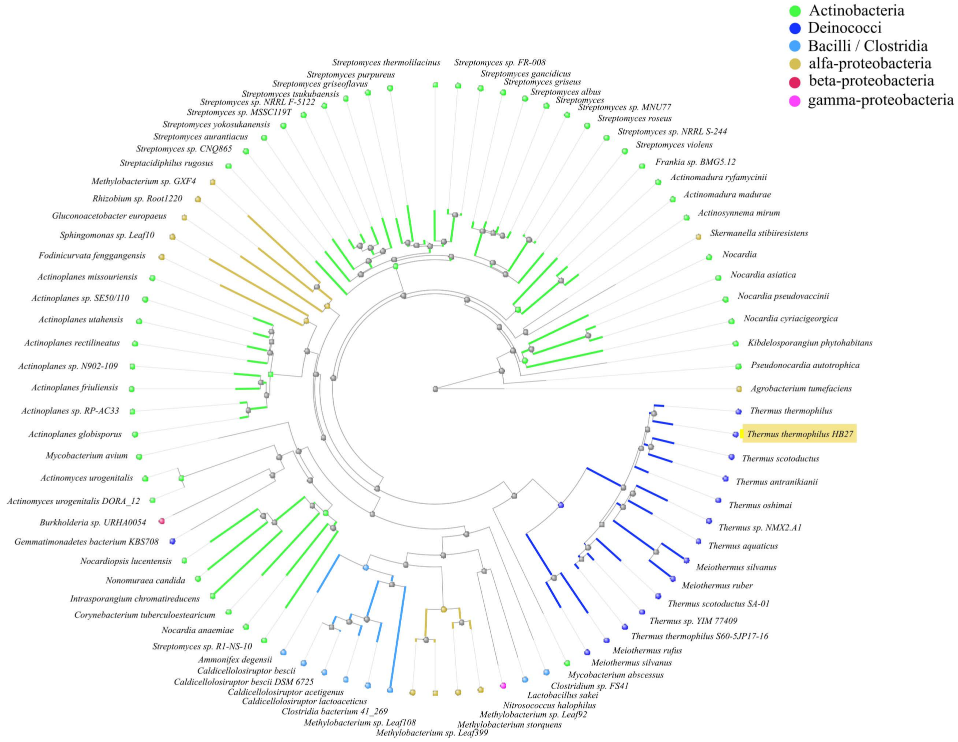

(c) A phylogenetic tree was generated at the BLAST web server, using the 90 bacterial sequences selected, and choosing the Fast Minimum Evolution method, with the following settings: maximal sequence difference: 0.85; distance: Grishin (protein). The tree was represented using the NCBI Tree Viewer application and selecting a Circular Tree layout, but emphasizing the evolutionary distance (indicated with bold/colored lines). The different groups of bacteria for which a potential TthPrimPol close orthologue has been detected, are indicated and differentially colored.

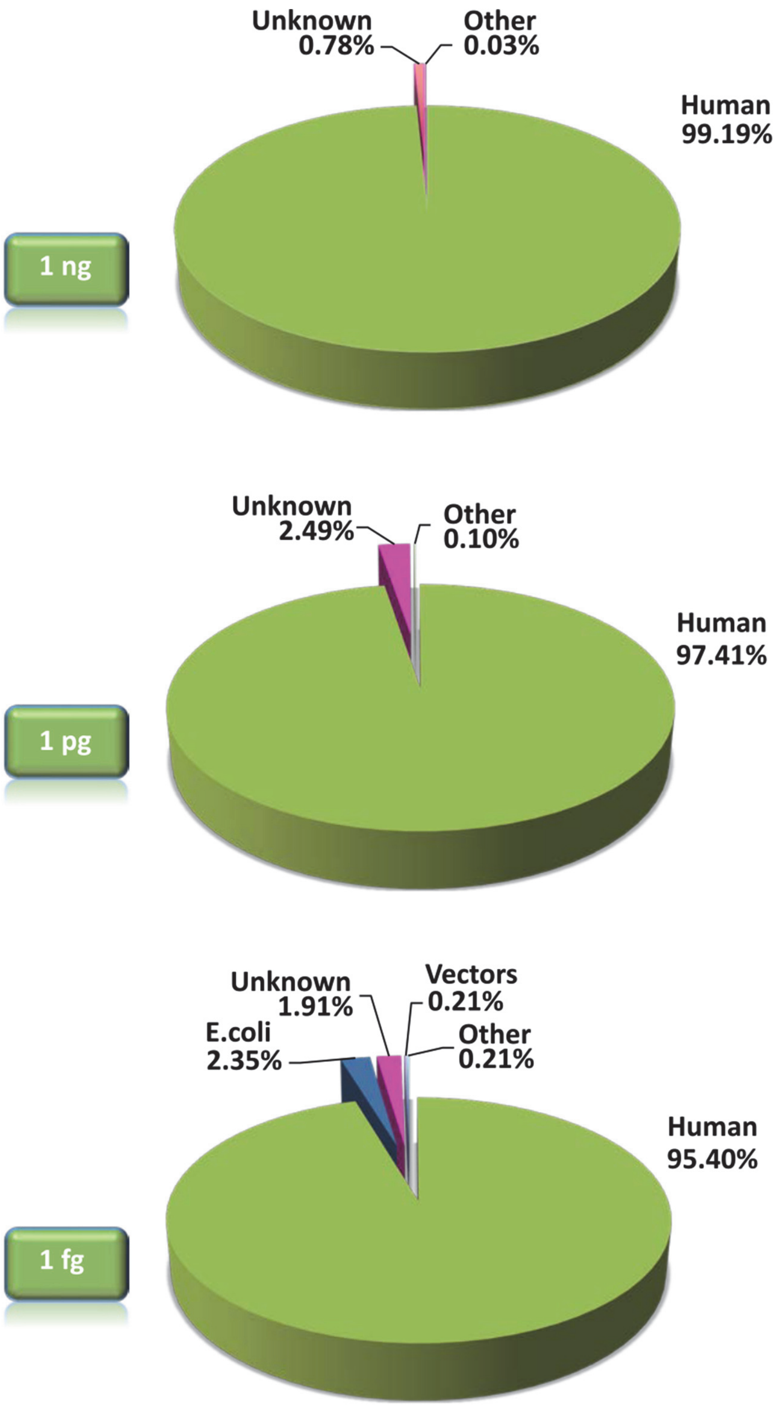

Supplementary Figure 2

TruePrime-amplified DNA is target derived even at 1 fg input. Diagrams showing the percentage of reads that can be assigned unanimously to the human genome. Input amount were 1 ng, 1 pg, and 1 fg of purified genomic DNA (Promega). Amplification was performed for 6 h. Sequences were obtained using the Ion Torrent platform. Even at 1 fg input more than 95% of the obtained reads are target derived.

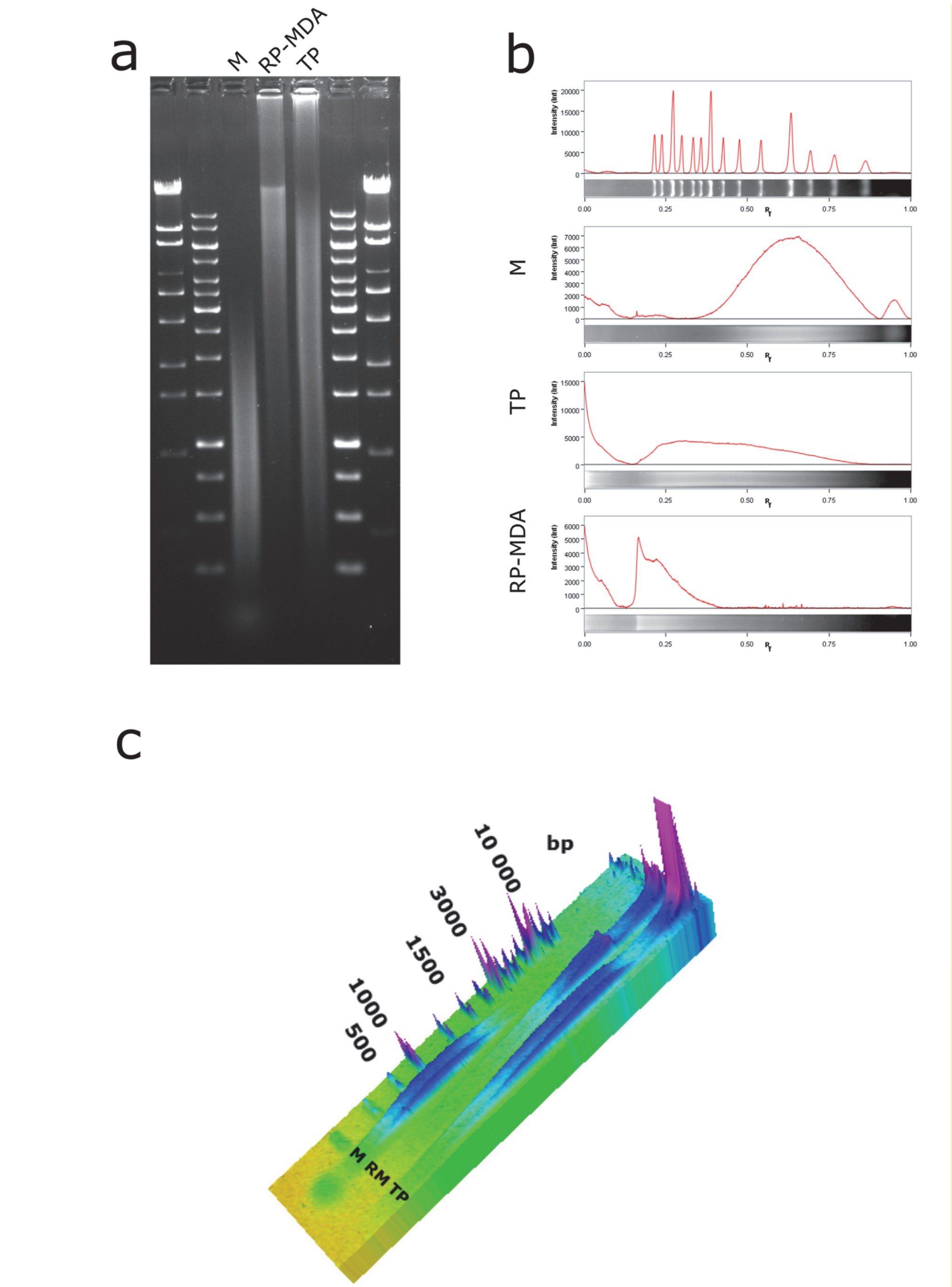

Supplementary Figure 3

Fragment sizes of amplification products for TruePrime, RP-MDA, and MALBAC. (a) Agarose gel (0.8%) electrophoresis of the amplification products (1 µg), M, MALBAC; TP, TruePrime; RP-MDA, commercial RP-MDA protocol; size markers lambda Sty and 2-log DNA ladder from NEB. **(b)** Densitometry of gel image. **(c)** Pseudocolor 3D graph showing size distributions.

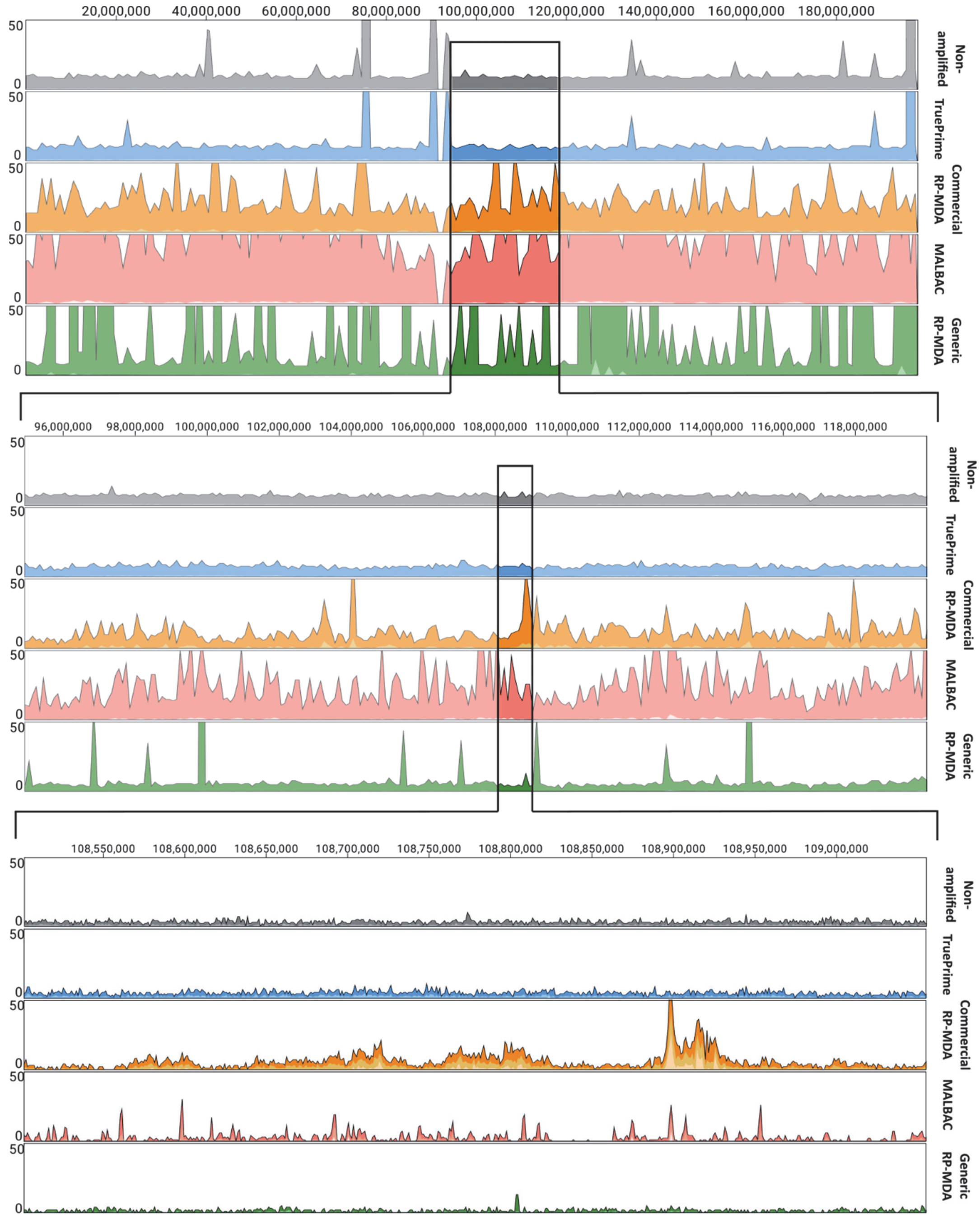

Supplementary Figure 4

Close-up views on chromosomal coverage between the different amplification protocols. Sliding window coverage comparison of exactly 12 million aligned read pairs of chromosome 3 between non-amplified (grey), TruePrime (blue), commercial RP-MDA kit (orange), MALBAC (red), and generic RP-MDA protocol (green). TruePrime shows a highly similar coverage pattern even in a window of only 450kb as compared to NA, as does the generic RP-MDA protocol, but with lower coverage. The commercial RP-MDA kit displays certain over-amplified regions, MALBAC shows regions without any amplification, which corresponds to the much lower coverage breadth.

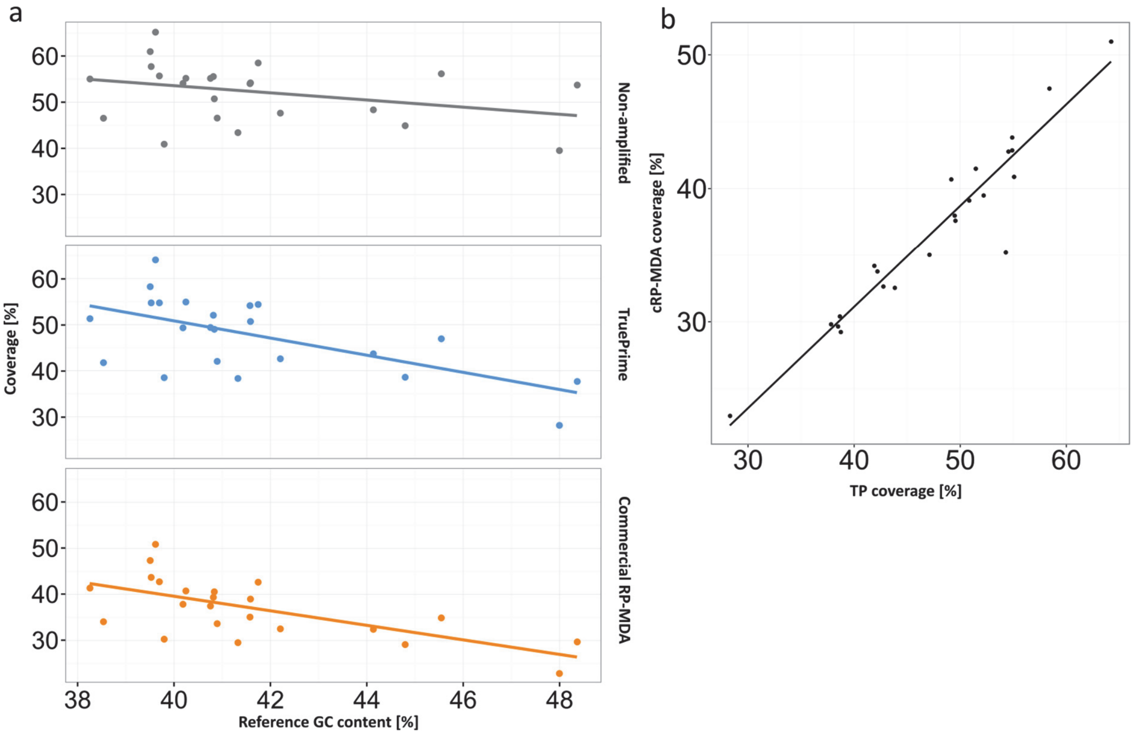

Supplementary Figure 5

Regression plots showing dependency of chromosomal coverage from GC content. (a) The notable difference between coverage of different chromosomes in the non-amplified sample (NA) is due to some other basic bias in the library prep or Illumina sequencing protocol as the effect of GC content on chromosomal coverage is not significant. **(b)** In the TruePrime-amplified sample (TP) there is a significant effect of GC content on chromosomal coverage (R^2=0.38; p=0.0017). **(c)** Surprisingly, the behavior of the commercial RP-MDA is identical to TruePrime in this regard (R^2=0.44, p=0.0006), implying that the main driver behind this GC-dependence in relative chromosomal coverage is Φ29DNApol, not the priming mechanism. **(d)** High correlation of the chromosomal coverage patterns between the two amplification methods (R^2=0.92).

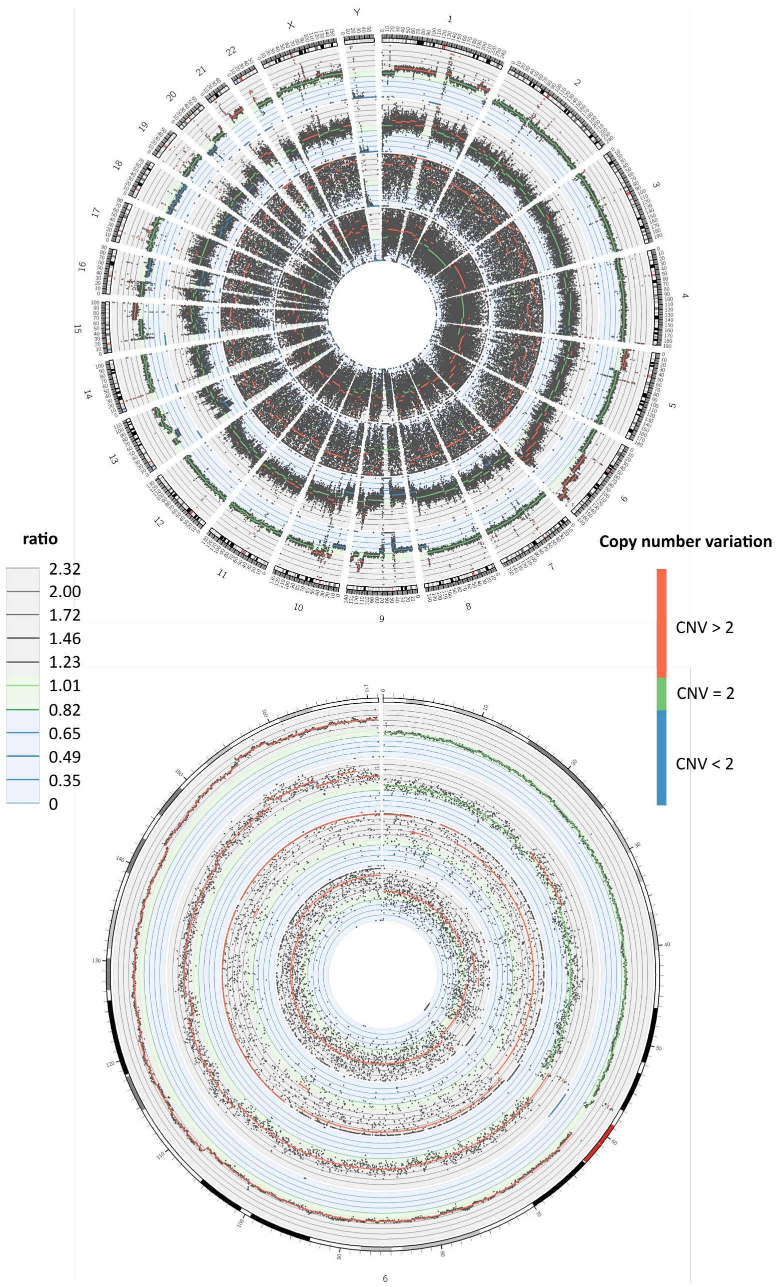

Supplementary Figure 6

CNV calling using ControlFreeC. From outward to inward: non-amplified, TruePrime, commercial RP-MDA kit, and MALBAC. The binned read depth (bin size = 50kb) is shown as black dots, whereas the calculated copy number is shown as a colored line (blue = one copy (haploid), green = two copies (diploid), and red = more than two copies (polyploid)). The top Circos plot shows all chromosomes, the bottom Circos plot a close-up of chromosome 6. The NA shows very little dispersion of binned read depths. TruePrime displays a greater dispersion, but allows for a largely identical calculation of ploidy states. The commercial RP-MDA kit shows a high fluctuation in binned read depth and often a very high calculated copy number. MALBAC displays a similar behavior, with better prediction of ploidy states than the commercial RP-MDA sample.

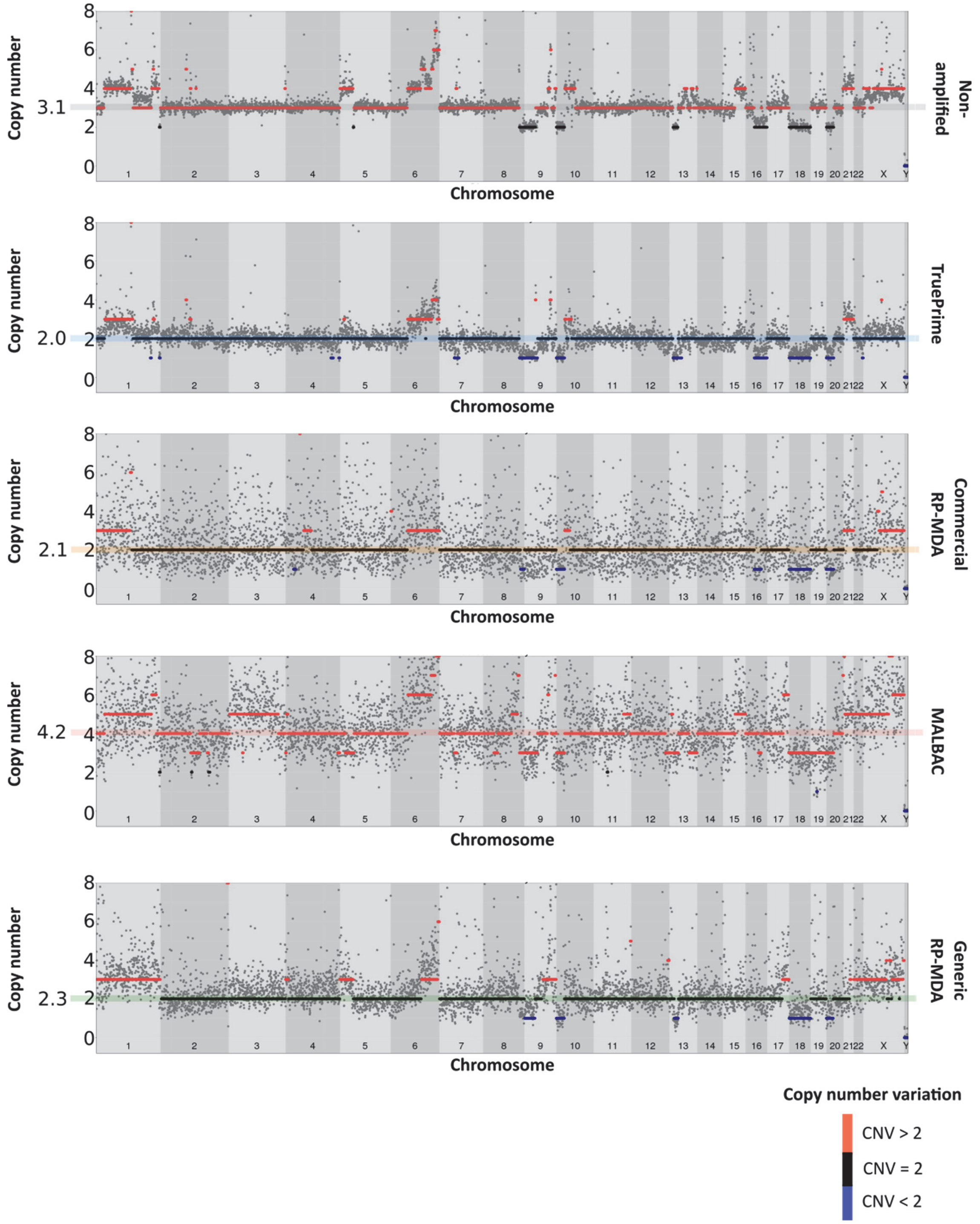

Supplementary Figure 7

CNV calling using Gingko. Shown are the read numbers per bin (dots), and the deduced ploidy level of the chromosomal segment (lines). TruePrime shows the lowest dispersion level of bins and is closest to the non-amplified profile. Variable bin sizes of about 500 kb were used.

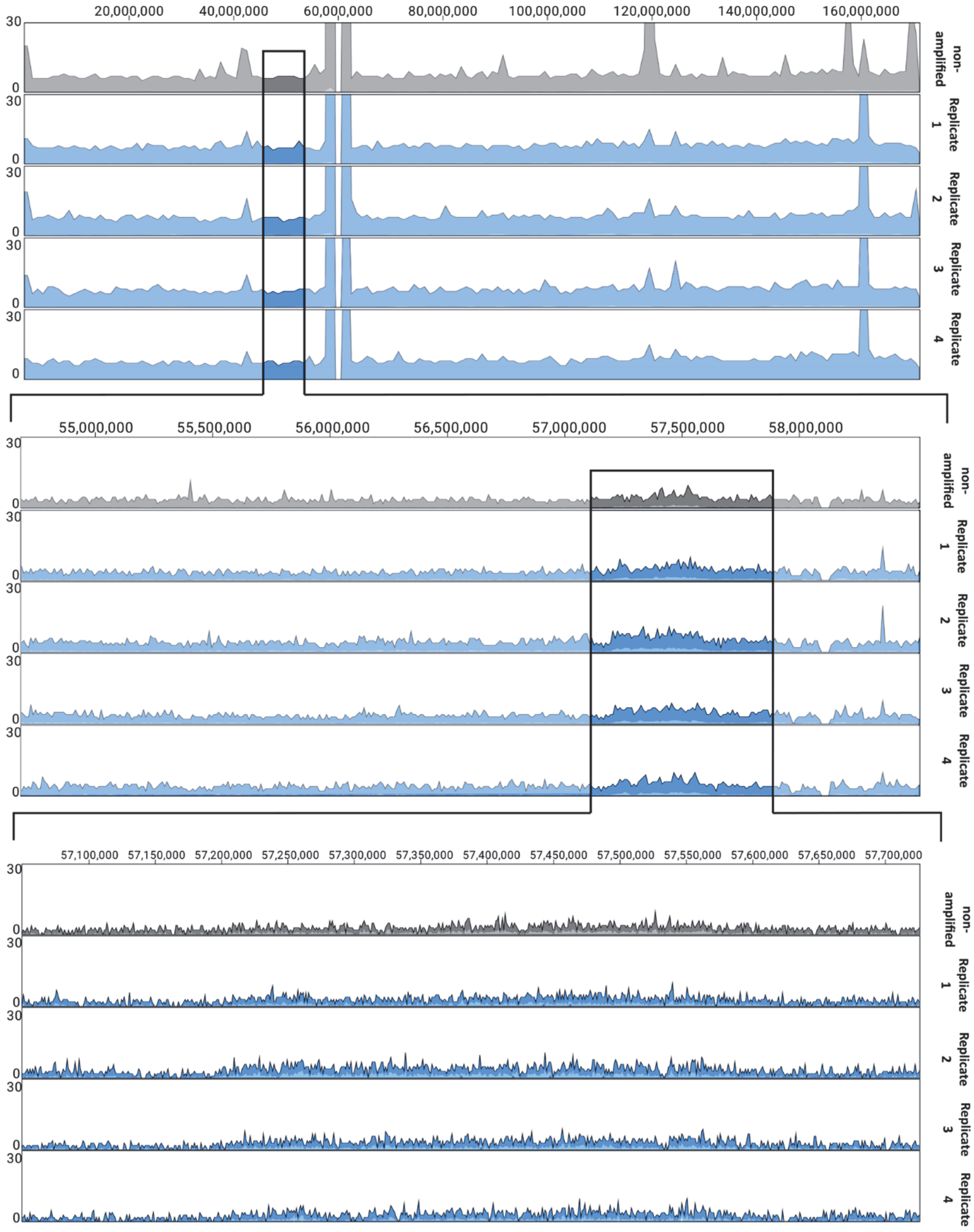

Supplementary Figure 8

Close-up views on chromosomal coverage reproducibility. Sliding window coverage comparison of chromosome 3 between non-amplified (grey) and four HEK293 cells amplified with TruePrime (blue) (input: exactly 5 million randomly selected read pairs). The coverage pattern between the four replicates and the NA sample is highly similar. Even a small region in the NA sample (around 57,500,000) which has a higher coverage than the surrounding region is reproduced by each TruePrime replicate down to a zooming region of only 600kb.

Supplementary Table

*Supplementary Table 1 - **SNV calling characteristics using 4 different variant callers.***

	NA	TP	RP-MDA	MALBAC		NA & TP	NA & RP-MDA	NA & MALBAC
# of SNV								
Isaac	2812735	2536654	1359855	2126935		2204003	1074975	540,192
samtools	4483645	4935242	3893398	9501504		3805174	2912703	2,039,595
VarScan	3079790	2822876	1749632	2319256		2550410	1487912	930,810
CLC lowfreq stringent	2967737	2622877	1546730	2775197		2298144	1261817	716,791
**Median**	**3023764**	**2722877**	**1648181**	**2547227**		**2424277**	**1374865**	**823801**
Fraction SNVs of NA								
Isaac	100.00%	90.18%	48.35%	75.62%		78.36%	38.22%	19.21%
samtools	100.00%	110.07%	86.84%	211.91%		84.87%	64.96%	41.33%
VarScan	100.00%	91.66%	56.81%	75.31%		82.81%	48.31%	32.97%
CLC lowfreq stringent	100.00%	88.38%	52.12%	93.51%		77.44%	42.52%	27.33%
**Median**	**100.00%**	**90.92%**	**54.46%**	**84.57%**		**80.58%**	**45.41%**	**30.15%**
Novelty rates SNV								
Isaac	6.03%	6.83%	13.52%	70.24%		5.58%	5.90%	5.76%
samtools	22.93%	31.20%	31.20%	78.91%		14.34%	11.42%	12.18%
VarScan	17.53%	18.22%	21.01%	56.80%		14.12%	12.70%	11.43%
CLC lowfreq stringent	9.26%	9.49%	13.88%	67.85%		7.64%	7.47%	5.32%
**Median**	**13.40%**	**13.86%**	**17.45%**	**69.05%**		**10.88%**	**9.45%**	**8.60%**
Het/Hom SNV								
Isaac	1.04	1.06	0.72	4.33		1.00	0.80	0.67
samtools	1.08	1.83	0.91	2.40		1.20	0.61	0.5
VarScan	0.81	0.77	0.39	1.38		0.64	0.27	0.33
CLC lowfreq stringent	1.03	1.00	0.62	3.05		0.98	0.49	0.4
**Median**	**1.04**	**1.03**	**0.67**	**2.73**		**0.99**	**0.55**	**0.45**
Ts/Tv SNV								
Isaac	2.06	2.03	2.04	1.12		2.07	2.07	2.1
samtools	1.74	1.57	1.82	1.09		1.82	1.90	1.86
VarScan	1.85	1.83	1.84	1.24		1.87	1.94	1.95
CLC lowfreq stringent	1.90	1.94	1.78	1.12		1.98	2.00	2.1
**Median**	**1.88**	**1.89**	**1.83**	**1.12**		**1.93**	**1.97**	**2.03**
Recall (position based)								
Isaac	50.42%	45.34%	22.70%	11.64%		-	-	-
samtools	57.36%	56.46%	46.47%	32.42%		-	-	-
VarScan	50.08%	45.82%	27.73%	18.03%		-	-	-
CLC lowfreq stringent	49.64%	44.22%	24.98%	15.88%		-	-	-
**Median**	**50.25%**	**45.58%**	**26.36%**	**16.96%**		-	-	-
Precision (position based)								
Isaac	67.11%	66.66%	63.86%	20.52%		-	-	-
samtools	56.81%	50.53%	50.16%	13.36%		-	-	-
VarScan	65.79%	65.41%	63.47%	31.04%		-	-	-
CLC lowfreq stringent	67.07%	66.97%	64.78%	21.81%		-	-	-
**Median**	**66.43%**	**66.04%**	**63.67%**	**21.17%**		-	-	-
conversion rate het -> hom (NA -> amplified) [%]								
Isaac	-	-	-	-		0.73%	21.91%	2.97%
samtools	-	-	-	-		5.60%	29.94%	35.43%
VarScan	-	-	-	-		8.42%	46.30%	40.96%
CLC lowfreq stringent	-	-	-	-		6.29%	29.36%	26.67%
**Median**				-		**5.95%**	**29.65%**	**31.05%**

estimated ADO rate [%]								
Isaac	-	-	-	-		1.45%	35.95%	5.77%
samtools	-	-	-	-		10.61%	46.09%	52.32%
VarScan	-	-	-	-		15.54%	63.29%	58.12%
CLC lowfreq stringent	-	-	-	-		11.84%	45.39%	42.11%
**Median**	-	-	-	-		**11.23%**	**45.74%**	**47.22%**
FPR								
Isaac	-	-	-	-		1.08%	0.92%	5.13%
samtools	-	-	-	-		3.66%	3.17%	24.14%
VarScan	-	-	-	-		0.88%	0.85%	4.49%
CLC lowfreq stringent	-	-	-	-		1.05%	0.92%	6.66%
**Median**	-	-	-	-		**1.06%**	**0.92%**	**5.89%**
conversion rate hom -> het (NA -> amplified)								
Isaac	-	-	-	-		0.25%	0.53%	0.51%
samtools	-	-	-	-		20.51%	12.79%	12.73%
VarScan	-	-	-	-		5.61%	3.18%	8.89%
CLC lowfreq stringent	-	-	-	-		4.21%	2.68%	3.88%
**Median**	-	-	-	-		**4.91%**	**2.93%**	**6.39%**
conversion rate hom -> het (NA -> amplified), Chr 18 only								
Isaac	-	-	-	-		0.13%	0.53%	2.29%
samtools	-	-	-	-		1.29%	2.22%	4.24%
VarScan	-	-	-	-		0.95%	4.19%	1.25%
CLC lowfreq stringent	-	-	-	-		0.13%	0.00%	0.89%
**Median**	-	-	-	-		**0.54%**	**1.37%**	**1.77%**

Shown is the number of SNVs detected by samtools / bcftools, varscan2, ISAAC, and the low frequency caller from CLC BIO, the number of recovered SNVs as percentage of the SNVs detected in the non-amplified (NA) sample, the novelty rates of the SNVs (not contained in dbSNP137), the ratio of heterozygote/ homozygote SNVs, the transition / transversion ratio (Ts/Tv) of detected SNVs, the recall rate (position based), the precision (position based), the conversion rate of heterozygote to homozygote SNVs from the non-amplified sample to the amplified sample, the estimated ADO rate, the false positive rate (FPR), the conversion rate of homozygous to heterozygous SNVs for the complete genome and for the haploid chromosome 18 only.

Supplementary References

1. Altschul SF, *et al.* Gapped BLAST and PSI-BLAST: a new generation of protein database search programs. *Nucleic Acids Res* **25**, 3389-3402 (1997).

2. Iyer LM, Koonin EV, Leipe DD, Aravind L. Origin and evolution of the archaeo-eukaryotic primase superfamily and related palm-domain proteins: structural insights and new members. *Nucleic Acids Res* **33**, 3875-3896 (2005).
